# Moebius Syndrome associated with syringomyelia: a case report

**DOI:** 10.31744/einstein_journal/2025RC0876

**Published:** 2025-04-07

**Authors:** Fernanda Kimie Yamamoto, Fernanda Marques de Deus, Renata de Araújo Monteiro Yoshida, Erasmo Barbante Casella, Romy Schmidt Brock Zacharias

**Affiliations:** 1 Hospital Israelita Albert Einstein São Paulo SP Brazil Hospital Israelita Albert Einstein, São Paulo, SP, Brazil.

**Keywords:** Moebius Syndrome, Facial paralysis, Syringomyelia, Infant, Newborn

## Abstract

Moebius Syndrome is a genetic condition that results in inability for facial mimicry due to palsy of cranial nerves VI and VII. Syringomyelia is characterized by a dilation of the central canal in the spinal cord, and is generally asymptomatic. Both disorders are diagnosed using imaging tests. A newborn with no facial movements upon physical examination was admitted to the delivery room of our hospital. A specialist confirmed the condition to be Moebius Syndrome. Further investigation using magnetic resonance imaging indicated that syringomyelia was associated with Moebius Syndrome. Given that syringomyelia is a silent myelopathy with a possible impact on the future health of the patient, careful analysis is recommended when diagnosing Moebius Syndrome. Few similar cases have been reported to date. Further studies are warranted to determine the actual association between these two conditions. Since syringomyelia diagnoses are often made when testing for other conditions, as in this study, neurological examinations of the brain should be expanded to include the spinal cord, in order to verify the presence of coexisting disorders.

## INTRODUCTION

Moebius Syndrome (MS) is a non-progressive congenital disease that affects the facial and abducens cranial nerves unilaterally or bilaterally. This syndrome manifests as facial paralysis. The following features may be present at different degrees: fixed eye gaze, eyelid ptosis, paralysis of the sideways (lateral) movement of the eyes, and difficulty in sucking and swallowing. The latter characteristic may be accompanied by a delay in speech development.^(1-3)^

Patients with MS may present with malformations of the orofacial structures and lower limbs, such as hypoglossia, low implantation of the auricle, syndactyly, and equinovarus foot. In 1998, Abramson et al. classified the most common clinical characteristics of MS into the so-called CLUFT: Cranial Nerves, Lower extremity, Upper extremity, Facial structural anomalies, and Thorax.^(4)^ Facial cranial nerves V, X, XI, and XII can also be concomitantly involved with MS.^(2,5)^

This syndrome is a rare disease that affects approximately 1 in 250,000 live births and has an equivalent incidence between sexes.^(1,6)^ The etiology of MS and the underlying genetic mutations have not been well described in the literature. Most cases are sporadic; however, familial occurrences have been reported on rare occasions. In these situations, MS was mainly reported among first-degree relatives.^(7)^

Currently, the prevalent medical theory regarding the etiology of MS is related to transient fetal vascular ischemia in the first trimester of pregnancy. This event inhibits blood supply to the brainstem, resulting in cranial nerve dysgenesis. Evidence from Brazil that supports this theory associates the increase in MS cases with the illegal use of misoprostol as an abortion drug.^(1,3,8)^

Syringomyelia is spinal cord dilation caused by the accumulation of cerebrospinal fluid in the central canal. This condition can be asymptomatic or cause neurological symptoms owing to compression of the nerve roots in the spinal cord. It is generally diagnosed incidentally when conducting imaging tests, such as magnetic resonance imaging (MRI), for the investigation of other pathologies. This myelopathy is rare and has been associated with Chiari malformation type 1.^(2,9)^

This paper reports an uncommon case of MS associated with syringomyelia.

## CASE REPORT

A 32-year-old healthy primiparous woman without complications during pregnancy and only hydronephrosis identified by fetal ultrasound (US) delivered a baby in September 2021 in our hospital. The gestational age of the newborn was 40 weeks and its birth weight was 3.080 kg.

The child was born via C-section, with Apgar scores of 8-9. Facial paralysis was identified during the initial examination, and the newborn was transferred to the neonatal intensive care unit for investigation and monitoring.

Examination revealed hypertelorism, a flat nasal base, absence of facial movements, micro- and retrognathia, low ear implantation, decreased ocular motricity, gingival hypertrophy, a small tongue, and syndactyly in the 3rd and 4th fingers of the right lower limb. The female genitourinary tract was typical, with the presence of a perineal cleft and an anterior anus.

A kidney and urinary tract US was performed following the fetal examination. Both kidneys had normal morphology and regular contours; however, intermittent dilation of the pyelocaliceal system without hydronephrosis was observed.

Initially, the patient exhibited signs of incoordination between sucking and swallowing when offered an oral diet, which led to the insertion of an orogastric tube. One week after the tube was introduced, the patient transitioned to a complete oral diet and began breastfeeding with the support of dysphagia and swallowing therapy.

Multidisciplinary assessment and investigation tests were carried out. Ophthalmological evaluation revealed the presence of mild bilateral epiblepharon and the absence of abduction by the vestibulo-ocular reflex, and genetic evaluation confirmed the diagnosis of MS. The patient had a normal karyotype (46, XX). Neurological evaluation was also conducted.

**Figure 1:**
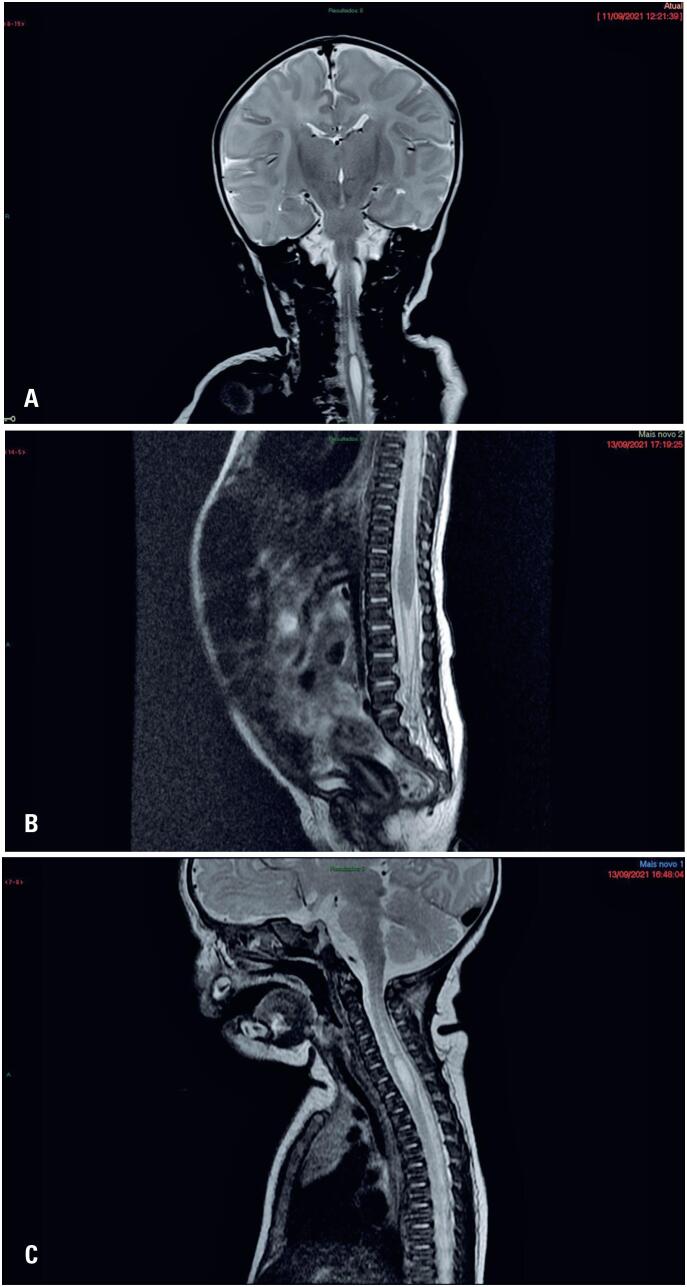
Incidental findings of cerebral and spine MRI. (A) Brain MRI - coronal T2, cervical; (B) Spine MRI - sagittal T2, lumbar thoracic; (C) Spine MRI - sagittal T2, cervical

A cerebral MRI performed without anesthesia revealed the absence of facial nerves, no evidence of facial colliculus, some flattening of the floor of the fourth ventricle, an uncharacterized abducens nerve, and signs of pons hypoplasia with reduced length in relation to that of the midbrain. Discrete enlargement of the cerebrospinal fluid space in the anterior aspect of the left middle cranial fossa and persistence of the cavum septum pellucidum were also observed. Cervical syringomyelia was found incidentally (Figure 1A).

Thereafter, a spinal MRI was performed (Figures 1B and 1C); it showed cervical syringomyelia and a thoracic spinal cord with apparent internal septations exhibiting greater anteroposterior caliber in the C7 plane. The rest of the spinal cord demonstrated normal caliber and signal strength. Brainstem Evoked Response Audiometry revealed no abnormalities.

The newborn was discharged 18 days after birth with advice of multidisciplinary follow-up.

This study was approved by the Research Ethics Committee of *Hospital Israelita Albert Einstein* (CAAE: 58441622.2.0000.0071; # 5.488.916). Informed consent was obtained from the parents of the patient.

## DISCUSSION

Moebius Syndrome is a rare condition that affects cranial nerves VI and VII, resulting in facial paralysis and an inability to abduct the eyes.^(1)^ Similar to the literature, this case report presents paralysis of cranial nerves VI and VII, a small structural malformation in the right lower limb, and associated syringomyelia.^(2)^

The clinical features of MS characterize a phenotype among patients with this syndrome. The clinical presentation of the patient in this study indicated the following characteristics that fit into the classification created by Abramson, expressed by the acronym CLUFT:^(4)^ involvement of cranial nerves VI and VII, micrognathia, and syndactyly.

A decrease in ocular motricity can be deduced from a fixed eye gaze and incomplete closing of the eyes. These ocular manifestations implicate the involvement of cranial nerve VI, which is responsible for the innervation of the homolateral lateral abductor rectus muscle.^(10)^ Syndactyly is a common feature in patients with MS.^(11)^

Moebius Syndrome is often misdiagnosed as autism or mental disability owing to a lack of facial expression, eye contact, and difficulty in communication.^(12,13)^ Such misdiagnosis can lead to inadequate treatment, making the syndrome more challenging for patients. Early rehabilitation and parental support can benefit individuals with MS.^(1)^

In this study, in addition to the absence of cranial nerves VI and VII, brain MRI revealed flattening of the floor of the fourth ventricle and no evidence of facial colliculus. These findings match descriptions of all the patients with MS in a retrospective study published in 2019 by the University of Antioquia.^(6)^

This case report also presents an incidental finding of syringomyelia during a brain MRI scan. The term "syringomyelia" is often used interchangeably with "hydrosyringomyelia," which combines the original terms "syringomyelia" and "hydromyelia."^(14)^ Both terms describe the pathological dilation of the central channel of the spinal cord.

Occasionally, syringomyelia is symptomatic and manifests as pain. In most cases, this condition develops asymptomatically for many years and is discovered incidentally during imaging tests for other problems. The number of syringomyelia diagnoses has risen due to an increase in MRI requests.^(15,16)^ Literature on this condition indicates an association with Chiari malformation type 1, which is asymptomatic in most patients.

Little is known about the possibility of genetic involvement in MS and syringomyelia. This clinical case is important because of the limited number of similar cases described in the literature. During this study, only one publication that correlated with the observed conditions was found: a case report published in 2012 in the Journal of Child Neurology that describes, for the first time, an association between MS and syringomyelia in a 2-year-old.^(2)^

This case report also presents an association between MS and syringomyelia, adding to the literature. The question arises as to whether MRI performed solely on the brain for patients with MS may prevent the detection of silent spinal cord pathologies such as syringomyelia.

## CONCLUSION

Imaging tests for patients with Moebius Syndrome should be expanded to include the spinal region. Further studies are required to better understand the correlation between these two conditions.

## AVAILABILITY OF DATA AND MATERIALS

The data that support the findings of this study are available from Cerner. However, restrictions apply to the availability of these data, which were used under license for the current study and are not publicly available. However, the data are available from the authors upon reasonable request and with permission from the parents of the patient.
